# Global burden of childhood tuberculosis

**DOI:** 10.1186/s41479-016-0018-6

**Published:** 2016-11-24

**Authors:** Helen E. Jenkins

**Affiliations:** Department of Biostatistics, Boston University School of Public Health, Boston, MA 02118 USA

**Keywords:** Incidence, Mortality, Estimation, Drug resistance

## Abstract

In 2015, the World Health Organization (WHO) declared tuberculosis (TB) to be responsible for more deaths than any other single infectious disease. The burden of TB among children has frequently been dismissed as relatively low with resulting deaths contributing very little to global under-five all-cause mortality, although without rigorous estimates of these statistics the burden of childhood TB was, in reality, unknown. Recent work in the area has resulted in a WHO estimate of 1 million new cases of childhood TB in 2014 resulting in 136,000 deaths. Around 3% of these cases likely have multidrug-resistant TB and at least 40,000 are in HIV-infected children. TB is now thought to be a major or contributory cause of many deaths in children under five years old, despite not being recorded as such, and is likely in the top ten causes of global mortality in this age group. In particular, recent work has shown that TB is an underlying cause of a substantial proportion of pneumonia deaths in TB-endemic countries. Childhood TB should be given higher priority: we need to identify children at greatest risk of TB disease and death and make more use of tools such as active case-finding and preventive therapy. TB is a preventable and treatable disease from which no child should die.

## Background

In 1963, Edith Lincoln and Edward Sewell wrote in their seminal book ‘Tuberculosis in Children’: “At the present time the death rate from tuberculosis is markedly reduced in some areas and it is possible to look forward to the day when tuberculosis will no longer be a public health problem [1].” More than 50 years later, in 2015, the World Health Organization (WHO) declared tuberculosis (TB) to be the number one killer among infectious diseases [2].

The underlying reasons for this resurgence are complex. They include HIV, the oldest known sample of which was taken just three years before the publication of ‘Tuberculosis in Children’, although it was not identified as HIV until many decades later [3]. Another contributory factor, drug resistance, was already known about in 1963, although few could have foretold the devastating impact it would have on TB control. But what has the impact been on children? What proportion of the estimated 9.6 million new TB cases in 2015 [2] occurred in children? How many were HIV-infected or infected with drug-resistant TB? And how many died? We simply don’t know many of these basic statistics, largely because the diagnosis of TB in children still relies heavily on the methods that Edith Lincoln was using in Bellevue Hospital more than half a century ago [1]. However, there is a growing understanding that many cases of TB disease in children are not reported as such [4].

While TB is the number one infectious cause of death among all age groups, pneumonia bears that title among children under five years old, with an estimated 935,000 deaths in 2013 [5]. *Mycobacterium tuberculosis*, the causative agent of tuberculosis, is also a recognized, although under-diagnosed, cause of pneumonia, especially in TB-endemic areas and among HIV-infected children [6]. A recent review found that between 1% and 23% of pneumonia cases also had TB disease [7]. The true extent to which tuberculosis is an underlying cause of morbidity and mortality attributed to other causes is unknown, largely due to problems with the diagnosis of TB in children [4]. However, with increasing attention on childhood TB in recent years [8], there have been considerable steps taken to use mathematical and statistical tools to help us understand the true burden of childhood TB.

Here, I summarize our current knowledge about the burden of childhood TB, with specific reference to incidence and mortality, as well as the impact of HIV and drug resistance on this vulnerable and often neglected population.

## Why is it important to understand the burden of childhood TB?

Firstly, we must consider what we mean by the word “burden”. Burden is a non-specific term measuring the impact of a health problem in terms of financial cost, mortality, morbidity or other indicators and here we will focus primarily on morbidity and mortality due to varying forms of TB. The reasons behind the need to understand disease burdens are broadly similar across diseases: without robust estimates of the true burden of disease, we cannot identify gaps in identification of cases, estimate the resources required to reduce this burden, begin to plan the types of interventions that might be effective or measure the impact of these interventions. The specific reasons for understanding the burden of childhood TB have been covered previously [9] but include the need to raise advocacy for childhood TB, which has been traditionally much neglected [10]; a need for increased research into improved diagnostics and treatment regimens specifically for children; demonstration of the importance of TB in the context of overall childhood morbidity and mortality; and also because childhood TB is a surveillance indicator for recent transmission within a community [11].

The END TB Strategy from WHO has the specific aims of reducing global TB incidence and mortality by 90% and 95% respectively by the year 2035 [12]. However, without good estimates of incidence and mortality it will be impossible to know if these targets have been met. Children make up 26% of the global population and 43% of the population in low-income countries [13]. Therefore, to monitor our progress towards the END TB goals, we need robust estimates of incidence and mortality in children.

## Incidence of childhood TB disease

In 2011, the WHO produced their first estimate of global childhood (<15 years) TB annual incidence of 490,000 [14], assuming equal case detection rates in children and adults. Earlier estimates had included 663,990 [15] (1990), 884,019 [16] (2000) and 1,039,000 [15] (2000). In 2014, three new pediatric TB incidence estimates were put forward. Jenkins et al. published an estimate of approximately 1 million [17] (Table 1). This was produced by scaling up age-disaggregated smear positive notifications reported to the WHO, in such a way as to account for the substantial difference that exists between adults and children in terms of the proportion of all TB cases expected to be smear positive [18, 19]. In the second method, Dodd et al. used a mathematical model that estimated the incidence of TB infection in children using WHO TB prevalence data and demographic information [20]. Their model then estimated childhood TB disease incidence by incorporating the age-dependent risk of progression from infection to disease, accounting for HIV infection and Bacille Calmette-Guerin vaccination. They estimated that there were 651,000 incidence cases of childhood TB in the 22 high-burden countries (HBCs) [20] in 2010. This was later updated to produce a global estimate of around 850,000 [21] in 2014 (Table 1). A third independent group, the Institute of Health Metrics and Evaluation (IHME), estimated that there were 150,000 incident pediatric TB cases in 2013 [22] (among HIV-negative cases only). Notably, this was lower than the number of cases notified by countries to the WHO.

**Table 1:** Annual estimates of the childhood tuberculosis (TB) burden among children aged 0-14 years

Measure	TB disease	Fraction of all pediatric TB cases	Latent TB infection
TB incidence	999,792 (95% CI: 937,877 – 1,055,414)^a^		
	847,000 (IQR: 558,000 – 1,280,000)^b^	N/A	67 million (IQR: 52.3 million – 85.7 million)^b^
	1,000,000 (UI: 900,000–1,100,000)^c^		
	150,000^d^		
INH-R TB	120,872 (95% CI: 96,628–149,059)^c^	12.1% (95% CI: 9.8%–14.8%)^c^	
	84,000^be^	9.9%^b^	6.8 million^b^
MDR-TB	31,948 (95% CI: 25,594–38,663)^a^	3.2%^a^	
	24,800 (IQR: 16,100–37,400)^b^	2.9% (IQR: 2.7%–3.1%)^b^	2.0 million (IQR: 1.6 million–2.6 million)^b^
Mortality	136,000 (range: 115,000–157,000)^c^	13.6%^c^	N/A
	60,000^d^		

*INH-R* Isoniazid resistant, *MDR* Multidrug-resistant, *CI* Confidence Interval, *IQR* Interquartile range, *UI* Uncertainty Interval

^a^Jenkins et al. 2014 [17]; ^b^Dodd et al. 2016 [21]; ^c^WHO 2015 [2]; ^d^Murray et al. 2014 (Note: there are no uncertainty ranges around these estimates); Yuen et al. 2015 [35]

^e^Note that Dodd et al. 2016 produced an estimate of INH-R TB mono-resistance of 58,300 (IQR: 38,300 – 87,000) cases. The figure presented of 84,000 represents all forms of INH-R (i.e. mono-resistance plus MDR-TB) but because this was not formally estimated in Dodd et al. 2016, there is no IQR around this result

Following a meeting of the WHO Global Task Force on TB Impact Measurement in 2015 [23], it was recommended that the WHO combine the methods of Jenkins et al. [17] and Dodd et al. [20] to produce their pediatric TB incidence estimates [23]. The method of Murray et al. was excluded due to lack of information about the uncertainty of the estimate [24]. The new WHO combined estimate was 1 million incident child TB cases in 2014 [2] (Table 1).

Given that only 359,000 pediatric TB cases were notified to the WHO in 2014, this implies that two-thirds of all children that developed active TB disease in 2014 were not notified. The assumption is that these children were not diagnosed and therefore did not receive treatment. The morbidity and mortality implications of so many children not receiving treatment are profound and very worrying. Estimation of how many of these “invisible” children exist has been an essential part of raising advocacy for these children and demonstrating the need for improved diagnostics [25] and methods to find these children (e.g. active case-finding [26]). TB is both preventable and treatable but we need to identify these children in the first place.

Of particular concern are children under the age of five years. These children are less likely to be diagnosed with TB, given that they have the disease, but more likely to suffer serious sequelae such as TB meningitis [27]. So far, Dodd et al. are the only group to specifically estimate TB incidence in children under five, although WHO under-five estimates may be published in their annual TB report later in 2016. Dodd et al. estimated that 51.4% of all pediatric cases of TB disease in 2014 occurred in children under five years of age [21] (Table 2). Applying this to the WHO pediatric TB incidence estimate would indicate that 514,000 children under five years of age developed TB disease in 2014, which is nearly four and half times the number notified to the WHO for that year [28].

**Table 2:** Annual estimated burden of childhood tuberculosis (TB) among children aged 0–4 years^a^

Measure	Estimate (Interquartile range)	Fraction of all pediatric TB (0–14 years of age)
TB incidence	435,000 (278,000–651,000)	51.4%
Isoniazid-resistant TB	42,200^b^	50.2%
Multidrug-resistant TB	12,700 (8,020–19,000)	51.2%

^a^All estimates are from Dodd et al. 2016 [21]

^b^Note that Dodd et al. 2016 produced an estimate of INH-R TB mono-resistance of 29,500 (IQR: 18,800 - 44,300) cases. The figure presented of 42,200 represents all forms of INH-R (i.e. mono-resistance plus MDR-TB) but because this was not formally estimated in Dodd et al. 2016, there is no IQR around this result

## Drug-resistant TB disease

Drug-resistant TB is under-diagnosed among all age groups due to the resources and costs required for diagnosis and limited access to testing facilities in many parts of the world [29]. Difficulties with obtaining bacteriologically positive sputum from children with TB only serve to amplify these issues [30]. It is believed that the majority of cases of pediatric drug-resistant TB are undiagnosed as such and therefore inappropriately treated [11], if they receive any treatment at all. With so much under-diagnosis of pediatric drug-resistant TB, how do we know how many children globally develop active TB disease annually due to a drug-resistant strain?

Until 2014, no estimate of the global burden of multidrug-resistant (strains resistant to the drugs isoniazid and rifampicin, the backbone of TB therapy) TB (MDR-TB) existed. A systematic review of the literature published before 12 January 2012 identified 97 reports that included 8,382 children with drug-susceptibility results for isoniazid and rifampicin [17]. Of these, 348 children tested positive for MDR-TB. In 2012, authors from the WHO reported results from an analysis of their full database of the Global Project on Anti-tuberculosis Drug Resistance Surveillance [31] on MDR-TB reporting between 1994 and 2011. They found that of 6,070 children tested for MDR-TB, 456 were positive. A 2012 review of the literature published before 31 October 2011 on treatment outcomes among children with MDR-TB identified 315 children with MDR-TB [32]. These three studies taken together indicate that, even assuming no overlapping of study populations, just 1,119 children with MDR-TB have been documented in the published literature.

In 2014, the first global estimate of the annual incidence of pediatric MDR-TB was published [17]. This work reviewed the literature for studies that included both children and adults tested for MDR-TB in the same setting and quantified the relationship between the percentage of new (treatment-naïve) adult TB cases with MDR-TB and the percentage observed in children. The authors then used WHO national estimates of the percentage of new TB cases with MDR-TB by country to estimate the percentage of childhood TB cases that had MDR-TB for every country worldwide. They then multiplied these percentages by their aforementioned pediatric TB estimates to obtain the global estimated number of pediatric MDR-TB cases in 2010 of 32,000 [17] (Table 1), i.e. 3.2% of total childhood TB incidence.

Although outcomes for children with MDR-TB who receive appropriate treatment can be excellent [32, 33], the vast majority of these 32,000 annual new cases are never correctly diagnosed with MDR-TB, much less receive appropriate treatment. It is sobering to think that the 1,119 children ever reported in the literature represent just 3.5% of the total incident cases that occur in one year.

The burden of other forms of drug-resistant TB also requires quantification. Isoniazid preventive therapy (IPT) is one of our most effective, but under-used, tools against pediatric TB [34]. However, its effectiveness could be undermined by isoniazid-resistant (INH-R) latent TB infection (LTBI). A recent review by Yuen et al. estimated that 12.1% of children with TB globally had INH-R TB disease (including mono-resistance and combined with other forms of resistance), representing 121,000 cases of disease [35] (Table 1). This percentage was highest in the former Soviet Union countries. Assuming that the percentage of TB cases with isoniazid resistance in children with active disease is reflected in those with LTBI, isoniazid preventive therapy will be ineffective in 12.1% of children with TB. It therefore crucial to understand which children might be at highest risk of INHR-TB so that other preventive methods can be used, such a regimen containing rifapentine or rifampicin [36].

Finally, Dodd et al. recently published an extension of their mathematical model to estimate the number of children with several different forms of drug-resistant TB [21]. Noting that their estimates worked from a lower baseline of overall TB incidence than those of Jenkins et al., they estimated that 24,800 had MDR-TB (i.e. 2.9% of all TB incidence) (Table 1) and 58,300 had mono-INH-R TB (i.e. 6.9% of all TB incidence). For comparison with the results from Yuen et al., the total INH-R TB resistance estimate from Dodd et al. was 84,000 cases constituting approximately 9.9% of all pediatric TB cases [21]. In addition, they estimated that 1,160 children had extensively drug-resistant TB (TB that is MDR-TB plus resistance to a fluoroquinolone and an injectable drug).

## TB incidence among HIV-infected children

Despite the known importance of HIV co-infection in TB infected and diseased individuals [37], there are no estimates of the global burden of TB specifically in HIV-infected children. Dodd et al. estimated that 5.0% (IQR: 2.4%, 10.1%) of TB incidence in the 22 HBCs occurs in HIV-infected children [20]. This translates to 32,500 HIV-infected children developing active TB disease in the HBCs in 2010. A back-of-the-envelope calculation would suggest that globally in 2014 between 40,000 and 50,000 HIV-infected children developed TB disease.

We currently have two effective ways to prevent TB disease in TB-infected children: isoniazid prophylaxis [38] and anti-retroviral treatment (Dodd et al. in preparation). Many of these 40,000–50,000 annual cases could be prevented with more rigorous deployment of these preventive measures.

## Childhood mortality due to TB

Globally, an estimated 6.3 million children under five years old died in 2013 [5] from all causes. But how many of these children are dying from TB? The first WHO estimates of TB mortality among children, released in 2015, found that 136,000 children under fifteen years old died in 2014 from TB [2] (Table 1). This estimate was based on data from vital registration systems and mortality surveys from 129 countries; an imputation method was used for the remaining countries without such data (largely from Africa). Vital registration death data have several limitations. For example, a cause might not be attributed [39] or if it is, it might be incorrect, especially if only one cause of death is allowed [40], despite multiple contributory causes. For example, studies have shown that TB may come close to bacterial pneumonia as a respiratory cause of death in children [41, 42]. In addition, in many countries there are few resources to carry out autopsies, and some deaths may not even be registered at all [43]. These limitations are likely to be amplified in high TB burden countries with few resources to carry out detailed autopsies. The IHME group also produced childhood TB mortality estimates of 60,000 TB-related deaths among HIV-negative children in 2014 [22].

An alternative method of estimating the number of children dying with TB is to multiply case fatality ratios (CFR; which could be defined in this context as the percentage of children that die within a year of a TB diagnosis) by the estimated incidence of pediatric TB. A recent systematic review and meta analysis has quantified CFRs among children with TB [44]. In particular, the authors searched the pre-chemotherapy literature to understand CFRs in children that do not receive TB treatment. The authors estimated that 21.9% (95% CI: 18.1%, 26.4%) of children from the pre-chemotherapy era studies died from TB, within a year of TB diagnosis. The case fatality ratio among children aged under 5 years old was substantially worse at 43.6% (95% CI: 36.8%, 50.6%) [44]. Children receiving treatment faired considerably better with less than 1% dying.

That nearly half of all children under five years of age that do not receive treatment will die should be a call to action. Mortality due to TB is likely a far more substantial problem than currently thought and we urgently need to find and treat these children to prevent unnecessary deaths. If we assume that all of the estimated children aged under five years with incident TB that were not notified to the WHO in 2014 did not receive treatment, our case fatality ratios would suggest that 173,000 of these children died. This is already substantially higher than the current childhood estimate of 136,000 and does not include children aged between 5 and 14 years old or account for any potential increased risk associated with HIV-infection.

Tuberculosis was not mentioned in a recent paper classifying the causes of global under-five mortality [5]. Assuming that 50% of the 136,000 deaths from TB, as per the WHO estimate, occur in children aged under five years, TB should have been classed as the ninth highest cause of death worldwide in children aged 1–59 months, above pertussis (Table in Liu et al. [5]). Our back-of-the-envelope estimate of 173,000 children would place TB at number six, ahead of meningitis, AIDS and measles.

The reality is that TB is causing disease in many more young children than we realize, resulting in undiagnosed, untreated TB and too many preventable deaths. TB is being misdiagnosed as other diseases and is also an underlying, undiagnosed cause of deaths that are attributed to other more easily diagnosed diseases, including pneumonia [4]. As Graham et al. [4] pointed out, if just 10% of the 935,000 currently attributed to pneumonia [5] were in fact due to TB, this would add a further 93,500 deaths to the WHO estimate of 136,000, increasing it by 69%. Full recognition of the contribution that TB is making to under-five mortality is a first and essential step in reducing that contribution.

## Meningitis

The main cause of serious morbidity and mortality in children with TB is TB meningitis [27]. The successes of the roll-out of pneumococcal vaccines in recent years [45, 46] have resulted in TB meningitis becoming one of the most prevalent forms of bacterial meningitis [47, 48]. There are currently no estimates of the number of children that develop TB meningitis worldwide or that die from the disease, largely due to difficulties with diagnosis [49]. However, a recent study found that 19.3% (95% CI: 14.0%, 26.1%) of children with TB meningitis will die and that among the survivors 53.9% (95% CI: 42.6%, 64.9%) will experience neurological sequelae [50]. Given the high mortality and morbidity associated with this form of TB, we urgently need to understand how many children develop and die from this disease and where they are most prevalent. This is a major gap in our knowledge regarding the global burden of childhood TB.

## Latent TB infection

One important way of preventing future morbidity and mortality due to TB is through active case-finding to identify probable cases of latent tuberculosis infection (LTBI) in children and target those children with preventive therapy [26]. However, we need robust estimates of how many children are likely to have LTBI and where these children are located so that we can maximize the effectiveness of our active case-finding. Dodd et al. estimated that 67 million children under fifteen years old were infected with TB in 2014 [21] (Table 1). The majority of these were located in the SouthEast Asia region (27 million) and the African region (20.9 million) [21]. In addition, Dodd et al. estimated the number of children latently infected with various forms of drug-resistant TB [21] (Tables 1 and 2). These estimates by Dodd et al. were generated assuming a constant annual rate of infection (ARI) and extrapolating backwards over 15 years. Houben and Dodd have recently produced an estimate of annual LTBI prevalence in children of 97 million [51]. In their method, the historical ARI was allowed to vary, based on changes in WHO estimates of TB disease prevalence and direct ARI estimates from tuberculin skin test surveys.

These are important statistics to know but it is unrealistic to think that all of these children can and should be given preventive therapy. Yuen et al. recently published estimates of how many children might be targeted for preventive therapy [52]. The authors estimated how many children live in a household with at least one adult diagnosed pulmonary TB case and, therefore, have been at risk of transmission and should be offered preventive therapy. The authors also estimated how many of these child contacts were likely to already have TB disease at the time that they are investigated. The result was an estimated 7.48 million children living with an adult diagnosed pulmonary TB case, of which, 2.41 million were under five years old. Of these 7.48 million, the authors estimated that approximately 660,000 would have TB disease upon investigation, with 239,000 aged under five years. National or sub-national targets such as these allow a national tuberculosis program to plan resources and interventions to identify and treat children at risk of or already experiencing TB disease.

## Conclusions

Focus on and development of methods to estimate the global burden of childhood TB have advanced enormously in recent years; childhood TB is starting to get the recognition that it unfortunately deserves, although much more could be done. Approximately 1 million children develop TB disease each year and at least 14% die, probably considerably more. We are beginning to drill down and understand the burden among children infected with HIV, as well as the risk of drug-resistant forms of TB in children. The majority of these children are never diagnosed with or treated for their TB disease and TB is likely a far more important cause of under-five mortality than is currently believed. Now that we are beginning to appreciate the scale of the problem, we need a more accurate understanding of precisely which children are at greatest risk of morbidity and mortality so that they can be targeted with preventive treatment. No child should die from TB in the 21^st^ century.

## Abbreviations

ARI: Annual risk of infection
BCG: Bacille Calmette-Guerin vaccination
IHME: Institute of Health Metrics and Evaluation
INH-R TB: Isoniazid-resistant tuberculosis
IPT: Isoniazid preventive therapy
LTBI: Latent tuberculosis infection
MDR-TB: Multidrug-resistant tuberculosis
TB: Tuberculosis
WHO: World Health Organization
